# Case Report: A neurodevelopmental disorder with global developmental delay, microcephaly, eye anomalies, sweat dysregulation, and skeletal implications due to an ultra-rare *de novo* 5q14.3q15 copy number gain

**DOI:** 10.3389/fgene.2025.1549685

**Published:** 2025-05-26

**Authors:** Costela Lacrimioara Serban, Alexandra Mihailescu, Diana Miclea, Cristian G. Zimbru, Florina Stoica, Adela Chirita-Emandi

**Affiliations:** ^1^ Regional Center of Medical Genetics Timis, Clinical Emergency Hospital for Children “Louis Turcanu”, Part of ERN ITHACA, Timisoara, Romania; ^2^ Department of Functional Sciences, Discipline of Public Health, Center for Translational Research and Systems Medicine, “Victor Babes” University of Medicine and Pharmacy, Timisoara, Romania; ^3^ Department of Microscopic Morphology Genetics Discipline, Center of Genomic Medicine, “Victor Babes” University of Medicine and Pharmacy Timisoara, Timisoara, Romania; ^4^ Mother and Child Department, “Iuliu Haţieganu” University of Medicine and Pharmacy, Cluj-Napoca, Romania; ^5^ Medical Genetics Department, Clinical Emergency Hospital for Children, Cluj-Napoca, Romania; ^6^ Department of Automation and Applied Informatics, Politehnica University of Timisoara, Timisoara, Romania; ^7^ Center of Expertise for Rare Ocular Diseases, Emergency Clinical Municipal Hospital Timisoara, Part of ERN-EYE, Timisoara, Romania

**Keywords:** 5q14.3q15, iris hypoplasia, finger hypomobility, developmental delay, microcephaly, case report

## Abstract

This case report and literature review documents an ultra-rare *de novo* copy number gain at 5q14.3q15. The patient’s phenotype included hypotonia, microcephaly, global developmental delay, iris hypoplasia, atrophy, sweat dysregulation, and skeletal implications, including camptodactyly. This case presentation provides novel insights into the genotype–phenotype correlation for 5q14.3q15 copy number gain, particularly highlighting the involvement of the *MEF2C* gene (#MIM 600662). Through comprehensive clinical and genetic evaluation, we aim to enhance the understanding of this ultra-rare genetic condition and its implications.

## 1 Introduction

An ultra-rare condition is a medical disorder with an exceptionally low prevalence in the general population, typically affecting fewer than 1 in 50,000 individuals. These conditions are often poorly documented in the medical literature, making their diagnosis and management particularly challenging (Harari, 2016). Due to their rarity, ultra-rare diseases require a multidisciplinary approach and rigorous clinical evaluation to establish an accurate diagnosis and appropriate management strategies. Some have a distinct, nonetheless, non-specific phenotype that makes the clinical diagnosis difficult (Baynam et al., 2024; Bauskis et al., 2022). Copy number gain involving 5q14.3q15 is an ultra-rare condition characterized by a variable clinical presentation, which can include global developmental delay, physical anomalies, and other distinctive features (Le Meur et al., 2010; Cardoso et al., 2009; Novara et al., 2013).

The 5q14.3q15 region includes several genes with crucial roles in neurodevelopment and physical growth. Among these genes, *MEF2C* (#MIM 600662) is of particular interest due to its significant function in brain development and cognitive functions (Le Meur et al., 2010; Novara et al., 2013; Cesaretti et al., 2016; Yauy et al., 2019). Overexpression of *MEF2C*, whether due to 5q14.3 duplications, other copy number variations, or other factors leading to its overexpression, is believed to be responsible for many of the features associated with 5q14.3q15 copy number gain, including neurodevelopmental disorders and various physical anomalies. In contrast to deletions of the same region or loss of function variants in *MEF2C*, which are well-characterized and typically result in more severe phenotypes (Le Meur et al., 2010; Cardoso et al., 2009; Engels et al., 2009; Berland and Houge, 2010; Nowakowska et al., 2010; Zweier and Rauch, 2012), the region’s copy number gain appears to have fewer consequences. Still, they are very rare, and the understanding of the clinical picture and prognosis is less understood. This relationship between increased severity in segmental deletions and milder phenotype in duplications/copy number gains is similar to severity phenotypes described for other deletion/duplication scenarios such as 7q11.23 (Dentici et al., 2020) and 16p13.11 (Hannes et al., 2009).

Usually, 5q14.3q15 copy number gain syndrome arises *de novo*, and its clinical presentation can vary widely among patients, depending on the size of the gain, making diagnosis challenging. This variability underscores the importance of detailed genetic and clinical evaluation to better understand the genotype-phenotype correlation.

We aim to present a case report of a boy with a copy number gain at 5q14.3q15, initially diagnosed at 18 months and followed up until 9 years of age, showing a particular phenotype and intellectual disability. Additionally, cases presented in the literature are evaluated to further refine the genotype-phenotype relationship for 5q14.3q15 copy number gain.

## 2 Case description

The proband is a 9-year-old boy, born to a non-consanguineous couple, both parents being in their second decade of life (mother 27 years; father 28 years). He was the first child from two pregnancies, delivered at term by elective cesarean section. He had a birth weight of 2,820 g (−1.1 SD) and a birth length of 49 cm (−0.5 SD). The family history is unremarkable. He has a healthy brother.

### 2.1 Evaluation at 1 year and 6 months

**FIGURE 1:**
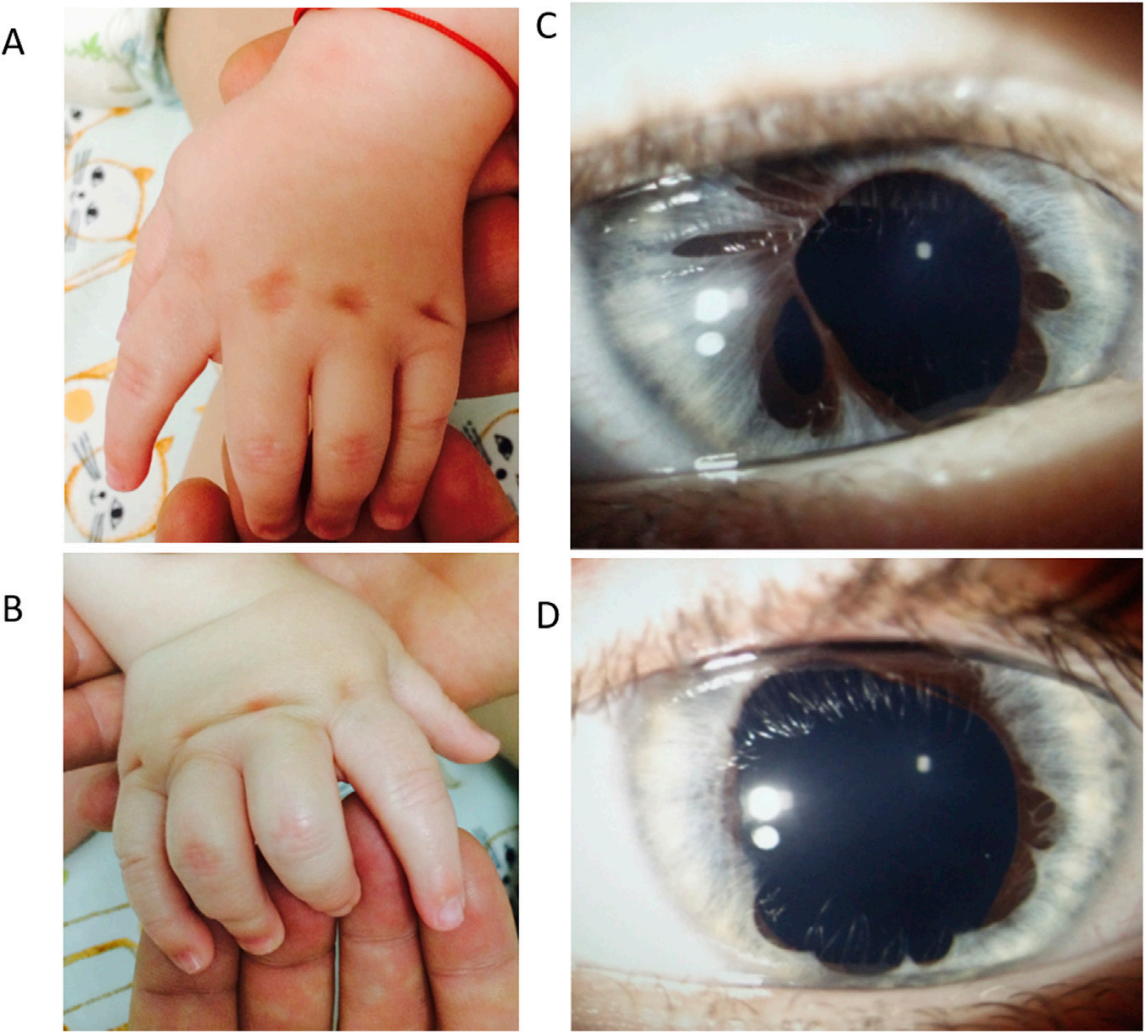
Patient’s phenotype at 1 year and 6 months. Panel **(A)**. Left hand, dorsal view. Cutaneous dimples over the metacarpophalangeal joint in fingers II–V. Panel **(B)**. Right hand, dorsal view. Fingers III-IV-V have camptodactyly and cutaneous dimples over the metacarpophalangeal joint in fingers II-V. Panel **(C)**. Anterior pole evaluation of the right eye: Irregular pupil, multiple iris atrophy areas. Panel **(D)**. Anterior pole evaluation of left eye: Irregular pupil, multiple iris atrophy areas.

At 1 year and 6 months, the boy presented with a particular facial phenotype: bilateral epicanthal folds, bilateral iris anomaly (iris hypoplasia/atrophy), convergent strabismus, turribrachycephaly, microcephaly, hypermobility of the lower limb joints, and right camptodactyly of fingers III-IV-V. Tapered fingers (thick at the base and thin at the tips) and bilateral talus valgus were also noted (Figure 1). Hypohidrosis was also reported by the parents. He had delayed motor skills, being able to sit unsupported at 10 months, and he could not walk independently at 18 months (achieved later at 22 months). His fine motor skills were impaired.

The ophthalmologic consultation noted bilateral iris dysgenesis/atrophy and concomitant strabismus in the right eye. He had normal visual acuity, and the retina was normal as assessed by optical coherence tomography (OCT). The cardiac evaluation and abdominal ultrasound were normal. The patient’s nerve conduction velocity test had normal results.

A brain MRI at age 8 months showed a 3-mm pineal cyst and minimal anomalies in the white matter (hypomyelinating lesions).

### 2.2 Diagnostic assessment

Clinical, laboratory, and genetic evaluations were conducted at the Romanian Regional Center of Medical Genetics Timis, which is affiliated with the European Reference Network ERN ITHACA. The ophthalmological evaluation was performed at the Center of Expertise for Rare Ocular Diseases, Emergency Clinical Municipal Hospital Timisoara, part of ERN-EYE, Timisoara, Romania.

#### 2.2.1 Genetic testing

**FIGURE 2:**
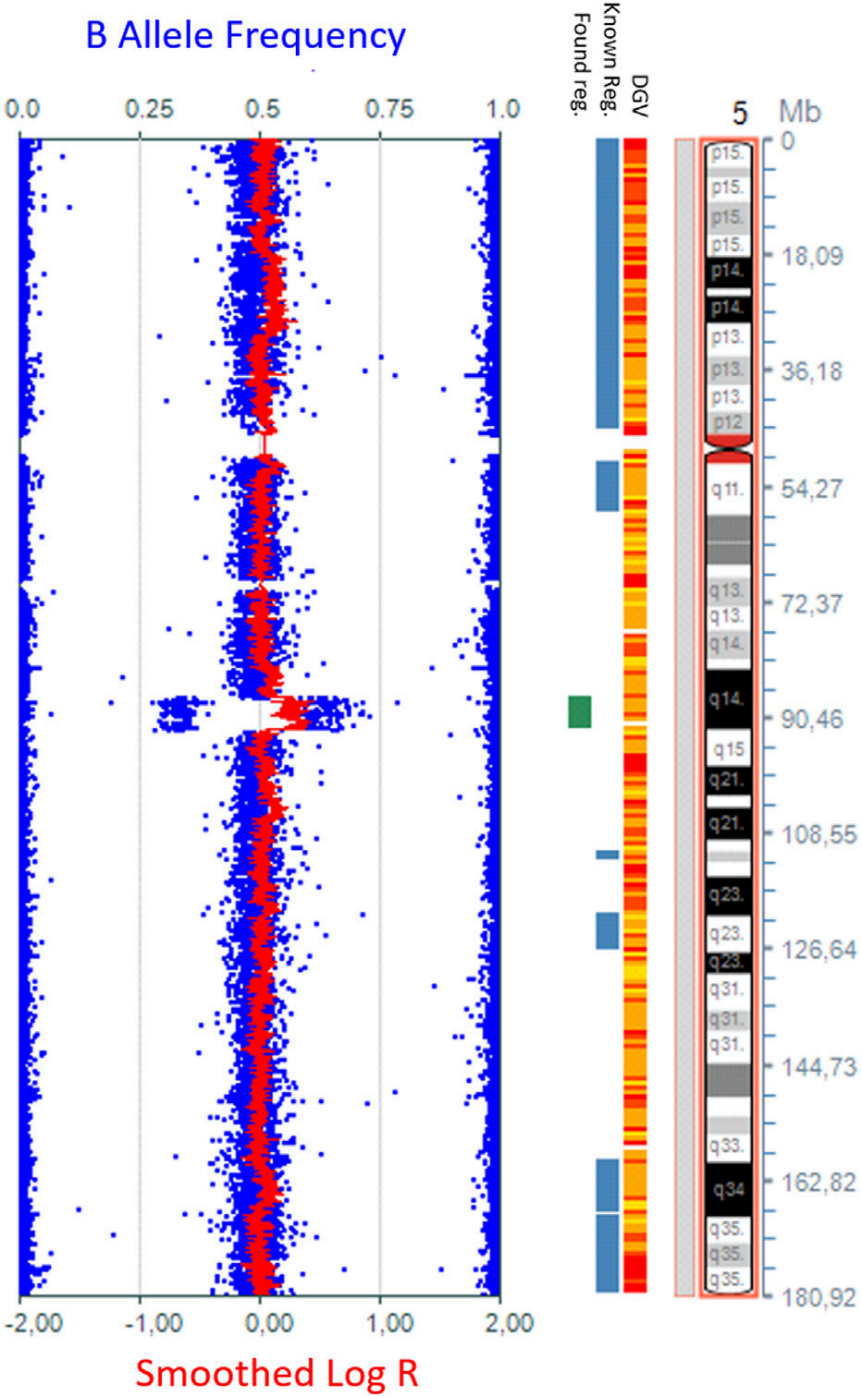
The 5.2-Mb copy number gain in the 5q14.3q15 region {arr [GRCh37] 5q14.3q15 (87269005_92546833)x3}, detected by whole-genome SNP-array analysis using KaryoStudio v1.4 software (Illumina, San Diego, CA) and an ideogram of chromosome 5.

Considering the global developmental delay and multisystem involvement, SNP-array analysis was performed for the child and parents. DNA extractions were performed with MagCore^®^ Automated Extraction Kits (RBC Bioscience Corp and quantified using a Qubit fluorometer. New Taipei City, Taiwan), using the blood protocol. High-density SNP-array analysis was carried out using the Infinium CytoSNP-850K v1.1 BeadChip kit (Illumina, San Diego, CA). The scanning was carried out using the Illumina iScan system (Illumina, San Diego, CA). The arrays were processed according to the manufacturer’s instructions using 200 ng of each DNA sample in a 4 μL volume. KaryoStudio v1.4 software (Illumina, San Diego, CA) was used for the primary analysis of the data generated by the scanner. B allele frequencies were used to determine copy numbers of chromosome regions in the abnormal chromosome represented in Figure 2. The SNP-array pattern (log R ratio and B allele frequency) was consistent with (from p arm to q arm) 2-3-2 copies of each region. The test detected a 5.2 Mb copy number gain on the long arm of chromosome 5:{arr [GRCh37] 5q14.3q15 (87269005_92546833)x3,dn}. The copy number gain identified in the patient was not detected in either of the parents using SNParray, which was subsequently performed for the parents. Thus, in the patient, the copy number gain of the long arm of chromosome 5 was *de novo*. The copy number gain was classified as a variant with likely pathogenic significance at the time of the results (2018) (Figure 2). The copy number gain (X3) was also confirmed by MLPA kit P395 (from MRC Holland), which includes the *MEF2C* gene.

The pathogenicity score of the 5q14.3q15 copy number gain was calculated to be 1.2, according to the ClinGen CNV Pathogenicity Calculator (Riggs et al., 2020), which indicates that the detected copy number gain has pathogenic significance in accordance with the American College of Medical Genetics and Genomics (ACMG) and the Clinical Genome Resource (ClinGen) 2020 guidelines (Riggs et al., 2020).

### 2.3 Follow-up evaluation at 9 years of age

The patient returned for follow-up at the age of 9 years, presenting with a weight of 23 kg (−1.5 SD), a height of 128 cm (−0.5 DS), and a head circumference of 49 cm (−2.8 SD). He continues to exhibit moderate developmental delay with an IQ of 49 on the Wechsler Intelligence Scale for Children (WISC) scale and a language acquisition disorder.

New symptoms observed include excessive sweating, particularly in cold environments, with onset at age approximately 7 years, while before this age, he had hypohidrosis and risk of overheating. He has diurnal and nocturnal urinary incontinence. Physical examination revealed turribrachycephaly, macrotia, straight eyebrows, bilateral iris dysgenesis, convergent strabismus, prominent ears, and a low-hanging columella. Additionally, he displayed camptodactyly in fingers III and IV of the right hand, yet ameliorated flexibility of fingers in the left hand, an asymmetrical thorax, and anteroposterior flattening of the thorax (Supplementary Figure S5). Furthermore, he experienced gait disturbances, behavioral issues such as anxiety and temper tantrums, and had fine motor disability (such as the inability to close a zipper or to write). He was diagnosed with a mild autistic spectrum disorder.

The sweat dysregulation was considered chronic, not related to infection or other acute disease, as it lasted for more than 2 years. Growth hormone, glycemia, thyroid, parathyroid, and adrenal assessments were normal. A cardiologic evaluation was normal. Central sweat dysregulation was suspected. The brain MRI was not reevaluated due to anesthesia risks that were not acceptable to the family. The patient’s medical management included kinesiotherapy and several types of cognitive, behavioral, and speech therapies. He is in follow-up with neurology, ophthalmology, orthopedics, psychology, and rehabilitation medical professionals.

## 3 Discussion

The copy number gain identified at 5q14.3q15 has limited understanding in the literature. This report provides a detailed description of this ultra-rare *de novo* copy number gain 5q14.3q15 and the associated phenotype of a patient coupled with a literature review in order to improve the understanding of the associated phenotype.

Triplosensitivity refers to a condition in which having an extra copy of a gene (a duplication) leads to a clinically significant effect. Triplosensitivity is typically assessed using curated databases such as ClinGen, which evaluates evidence from genetic studies, functional data, and clinical reports to assign a probability score. This score reflects the likelihood that a gene duplication results in a pathogenic phenotype (Collins et al., 2022). In our case, the copy number gain encompassed 26 genes (Supplementary Figure S4). Among these, *MEF2C* had the highest likelihood of triplosensitivity scored at 1, followed by *ARRDC3* (#MIM 612464) with a triplosensitivity probability of 0.84 (Collins et al., 2022). The triplosensitivity probabilities of other genes within this region were below the threshold of 0.68, which is associated with a benign outcome. A tripolosensitivity score above the threshold of 0.68 was linked to an odds ratio of ≥2 of deleterious effects, while for triplosensitivity scores exceeding 0.94, the reported odds ratio was ≥2.7 (Karczewski et al., 2020) (Supplementary Figure S5).

According to the Human Protein Atlas, the *MEF2C* gene is expressed in the cerebral cortex, skeletal muscle, and tongue. During the development of the human body, the *MEF2C* (Myocyte Enhancer Factor 2C) gene interacts with other genes in a multigene network, playing a crucial role in brain development, as well as the development of the heart, blood vessels, immune system, muscles, and face (OMIM, 2024). Recently, it has been shown that *MEF2C* interacts with *MECP2* and *CDKL5* (#MIM 300203), the main genes associated with Rett syndrome (Zweier et al., 2010). Mutations in *MEF2C* from the 5q14.3q15 microdeletion syndrome region are a frequent cause of severe intellectual disability and diminish *MECP2* and *CDKL5* expression. The presence of an extra copy of *MEF2C* is one of the mechanisms that lead to overexpression of the related protein. This may affect this carefully controlled network of genes, for instance, by causing the upregulation and overexpression of other genes in the network. This is thought to lie behind many features associated with 5q14 duplications (Cesaretti et al., 2016; Yauy et al., 2019). Xu et al. (2006) demonstrated that overexpressing Mef2c to mouse cardiomyocytes caused cardiomyopathy or predisposed transgenic mice to more fulminant disease following pressure overload. In cultured cardiomyocytes, *MEF2A* (#MIM 600660) or *MEF2C* overexpression induced sarcomeric disorganization and focal elongation. The overexpression of *MEF2C* could explain the central nervous system implication. The sweat dysregulation could, in theory, be caused by brain involvement with the overexpression of *MEF2C*. The other genes in the region have ubiquitous expression in tissues, yet at the moment, a causal relation is not established. Hyperhidrosis may be caused by a neuronal dysfunction of autonomous nervous system regulation, leading to a hyperactivity of the sympathetic nervous system or to abnormal central managing of emotions. Importantly, there is no dysfunction of the sweat glands themselves (Wohlrab et al., 2023).

According to OMIM (2024), the *ARRDC3* gene encodes Arrestin Domain Containing 3, a protein that plays a role in regulating cell surface receptor expression and signaling. *ARRDC3* is involved in the internalization and downregulation of G protein-coupled receptors (GPCRs), thereby modulating cellular responses to external signals. It is also implicated in regulating energy balance and metabolism, particularly in adipose tissue, where it influences insulin sensitivity and lipid metabolism. Mutations or dysregulation of *ARRDC3* can affect these pathways and have been linked to various disorders, including obesity, cancer, and metabolic syndromes (Batista et al., 2020; Wedegaertner et al., 2022). Upregulation of *ARRDC3* suppresses colorectal cancer progression by destabilizing the oncoprotein YAP (Shen et al., 2018). In the placenta, overexpression has been associated with preeclampsia (Lei et al., 2020) by suppressing cell invasion and tube formation. The impact of *ARRDC3* overexpression on the patient’s phenotype is not known.

The gene function reported in the literature does not appear to explain the eye phenotype, as the MEF2C gene is not expressed in the eye, nor has it been previously associated with eye malformation. However, in the report from Banka et al. (2010), the phenotype of the patients is similar and could potentially be explained by the overlapping region involved in the copy number gain. Nevertheless, the impact of a copy number gain cannot be fully explained only by evaluating single gene triplosensitivity in the region.

**FIGURE 3:**
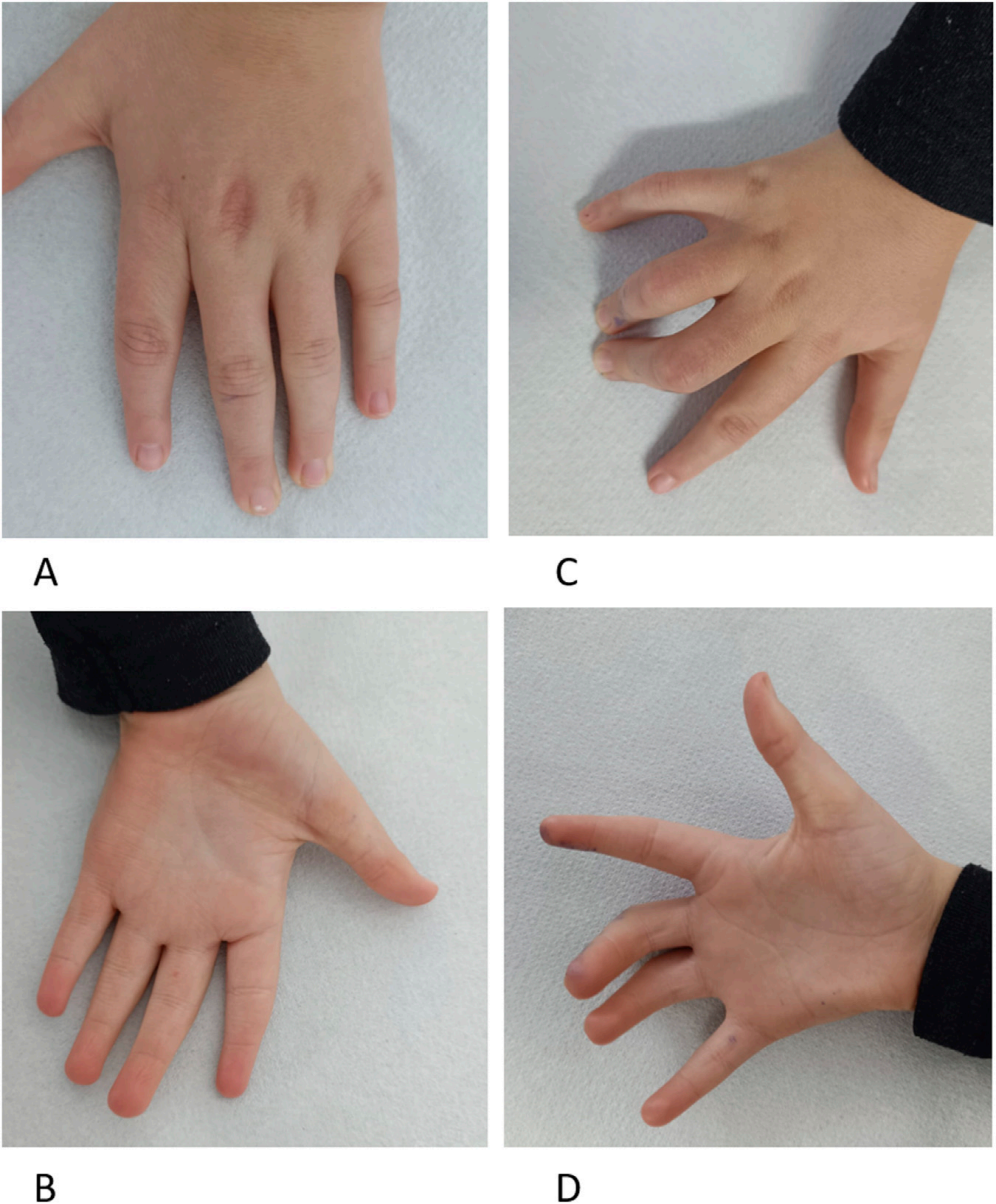
Patient at 9 years Panel **(A)**. Left-hand dorsal view with cutaneous dimples over metacarpophalangeal joints in fingers II–V and tapering fingers. Panel **(B)**. Left-hand palmar view with ulnar deviated fingers, tapering fingers, and unique palmar transversal crease. Panel **(C)**. Right-hand dorsal view with camptodactyly of fingers III-IV and cutaneous dimples over metacarpophalangeal joints in fingers II–V. Panel **(D)**. Right-hand palmar view with ulnar deviated fingers and unique palmar transversal crease, Fingers III–IV with camptodactyly.

At the time of this report, there are 25 cases in Decipher (Database of Genomic Variation and Phenotype in Humans using Ensembl Resources) that have overlapping copy number gain, of which 13 cases have copy number gain in 5q14.3 with overlap over the *MEF2C* gene (Figure 3). Of these, the copy number gain is considered pathogenic/likely pathogenic in seven cases. One case has a partial duplication of the *MEF2C* gene. The current case is Decipher Patient no 451461. Three cases from Decipher (Patients 401023, 307116, and 307966) have a relatively similar size and overlap over the *MEF2C, ADGRV1* (#MIM 602851), and *ARRDC3* genes, without additional copy number anomalies, similar to the patient presented here (see Table 1).

**TABLE 1:** Summary of the clinical features of 5q14.3 copy number gain involving the *MEF2C gene* (current patient compared to Decipher cases and literature (Le Meur et al., 2010; Novara et al., 2013; Cesaretti et al., 2016; Yauy et al., 2019; Banka et al., 2010)); also see Supplementary Table S1 for details.

Clinical description	Phenotype	This patient: Decipher patient ID 451461, ClinVarID 3775074	Number of people with feature/of total reported (not including the patient reported here)
Country, City of reporting laboratory		Romania, Timisoara	See supplementary table
Genotype GRCh38		5:87,973,188–93,211,127	See supplementary table
Gender	Male/female	Male	6/10 males
Age when reported	years	9 years	Range prenantal–10 years
Abnormality of the digestive system	Constipation	No	1/10
	Feeding difficulties	Yes	3/10
Abnormality of the ear	Macrotia	Yes	3/10
Abnormality of the eye	Deeply set eyes	No	1/10
	Strabismus	Yes	2/10
	Anterior pole anomaly	Yes	1/10
	Refractive errors	Yes	1/10
Abnormality of growth	Proportionate short stature	Yes	5/10
	Microcephaly	Yes	5/10
Abnormality of limbs	Broad Short thumb	No	1/10
	Tapered finger	Yes	1/10
	Fixed finger joints	Yes	0/10
Abnormality of the musculoskeletal system	Hypertonia	No	1/10
	Kyphosis	Yes	2/10
	Muscle weakness	Yes	3/10
	Pectus excavatum	No	1/10
	Head shape anomaly	Yes	4/10
	Scoliosis	No	1/10
Abnormality of the nervous system	Autistic behavior	Yes	4/10
	Delayed speech and language development	Yes	5/10
	Gait disturbance	Yes	3/10
	Intellectual disability	Yes, moderate	7/10
	Global developmental delay	Yes	7/10
	Seizures	No	2/10
	Brain malformation	No	3/10
	Anxiety	Yes	1/10
	Urinary incontinence	Yes	0/10
Abnormality of skin adnexa physiology	Hypohidrosis/hyperhidrosis	Yes	1/10
Cardiac anomaly	Ventricular hypertrophy	No	1/10

Additionally, two similar ClinVar reported copy number gains (Variation ID: 147564 and Variation ID: 58097) identified as pathogenic come from somatic testing and were not considered relevant for this report.

From this review of literature, the clinical phenotype of 5q14.3q15 copy number gain involving *MEF2C* gene included global developmental delay, delayed speech and delayed language development, mild or moderate intellectual disability, autistic behavior, microcephaly, head shape anomalies, and short stature as main characteristics. Other anomalies included anomalies of the eye (strabismus, anterior segment dysgenesis, and enophthalmia), finger anomaly, brain malformation, seizures, feeding difficulties, macrotia, pectus excavatum, and scoliosis. The presented child had anterior pole anomaly and hypohidrosis, followed by hyperhidrosis. These conditions were not previously reported in association with 5q14.3q15 copy number gain.

Based on the genotype–phenotype correlations observed in previous reports and the absence of other identifiable genetic or clinical diagnoses, we consider the newly described clinical findings to be most likely attributable to the 5q14.3q15 copy number gain. However, in the absence of functional studies, we acknowledge this as a limitation of our report. Other limitations are related to the fact that long-term outcomes remain unknown because our report covers the follow-up period from 18 months to 9 years. Further follow-up is needed to track the condition’s progression, particularly in cognitive and physical development. Additionally, the report lacks functional studies to confirm the mechanisms of gene overexpression, and experimental validation could better clarify the molecular pathways involved.

### 3.1 What is known?

• Copy number gain involving 5q14.3q15 is an ultra-rare condition characterized by a variable clinical presentation, which can include global developmental delay, intellectual disability, physical anomalies, and other distinctive features.

### 3.2 What does this report add?

• This report widens the molecular and clinical understanding of 5q14.3q15 copy number gain by showing a detailed eye phenotype (anterior pole anomaly) and sweat dysregulation not previously reported, together with a comparison with available phenotypic description.

## 4 Conclusion

Copy number gain involving 5q14.3q15 represents an ultra-rare condition characterized by a variable clinical presentation, which can include global developmental delay, malformation of the anterior pole of the eye, microcephaly, camptodactyly, failure to thrive, and sweat dysregulation.

## 5 Patient perspective

As parents of a child with an ultra-rare genetic disorder, we want to share the challenges that have shaped our family’s journey. We first noticed something was different when our son was 6 months old. He was not meeting typical growth milestones, which led us to seek medical advice. After numerous tests, we received a diagnosis that profoundly impacted our lives. Now 10 years old and in the second grade in Romania, our son struggles to keep up with his peers in school. His learning abilities are significantly behind; his IQ is only 49. He cannot write due to low fine motor skills and can only read small words. Focusing at school is difficult for him, and his physical development is also delayed, with poor muscle tone, motor skills, and vision issues from iris atrophy. He still has urinary incontinence day and night and must wear absorbent underpants. Despite these challenges, our son is a joyful and active child, even if he tires quickly. He is part of a local football team, where he enjoys being with his friends and learning teamwork. We have pursued numerous therapies to support his development, including horse therapy, hydrotherapy, speech therapy, kinesiotherapy, sound therapy, occupational therapy, and sensory integration therapy. While essential for his progress, these therapies are financially demanding. To help cover these costs, we established a small association to receive donations. Our son attends a mainstream school but requires daily support from an adult outside the school due to the lack of specialized resources in the Romanian educational system. Despite these obstacles, he brings immense joy to our family, and we remain committed to providing him with the best possible care to improve his quality of life.

## Data Availability

The data presented in the study are deposited in the Decipher (https://www.deciphergenomics.org/) repository, ID 451461 and ClinVar (https://www.ncbi.nlm.nih.gov/clinvar/) ID 3775074.

## Supplementary material

Supplementary Material Data sheet 1 Table S1 (xls) offers detailed clinical features of people with 5q14.3 copy number gain involving the MEF2C gene [current patient compared to Decipher cases and literature (Banka et al., 2010; Le Meur et al., 2010; Novara et al., 2013; Cesaretti et al., 2016; Yauy et al., 2019)].

Supplementary material Data sheet 2 S2 (Figures 4 and 5).
