# Supplementary material for: Case Report: A neurodevelopmental disorder with global developmental delay, microcephaly, eye anomalies, sweat dysregulation, and skeletal implications due to an ultra-rare de novo 5q14.3q15 copy number gain
Source: Front Genet. 2025 May 26;16:1549685. doi: 10.3389/fgene.2025.1549685 (PMC12146353; doi:10.3389/fgene.2025.1549685)
Supplement: Supplementary file 1 [file DataSheet1.docx]

Supplementary file

A neurodevelopmental disorder with global developmental delay, microcephaly, eye anomalies, sweat dysregulation and skeletal implications due to an ultra-rare de novo 5q14.3q15 copy number gain

Costela Lacrimioara Serban^1,2^, Alexandra Mihailescu^3^, Diana Miclea^4,5^, Cristian G. Zimbru^6^, Florina Stoica^7^, Maria Puiu^1^, Adela Chirita-Emandi^1,3^


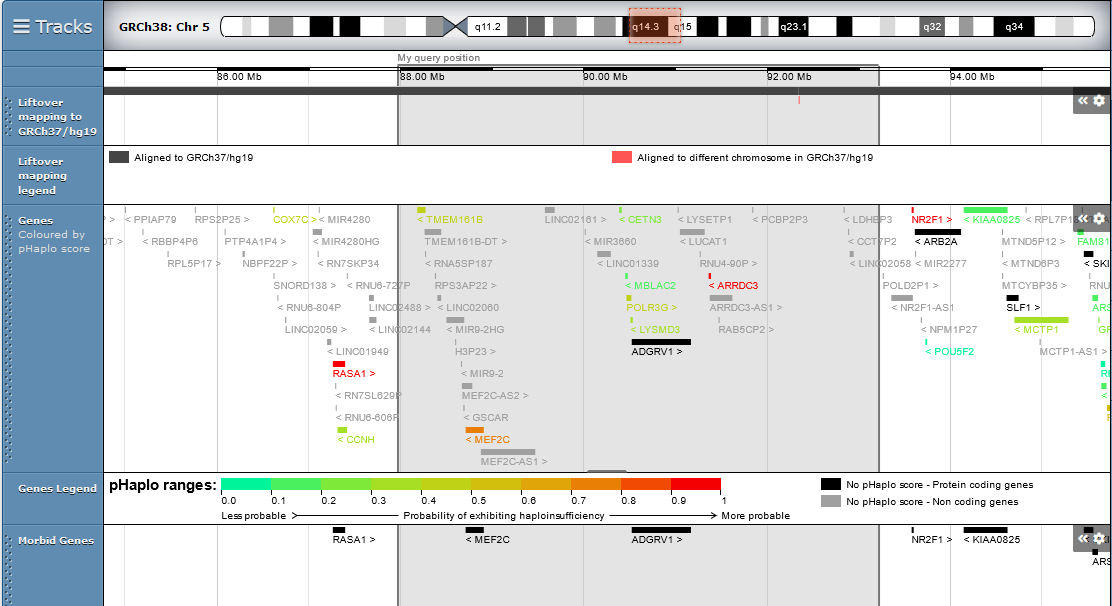


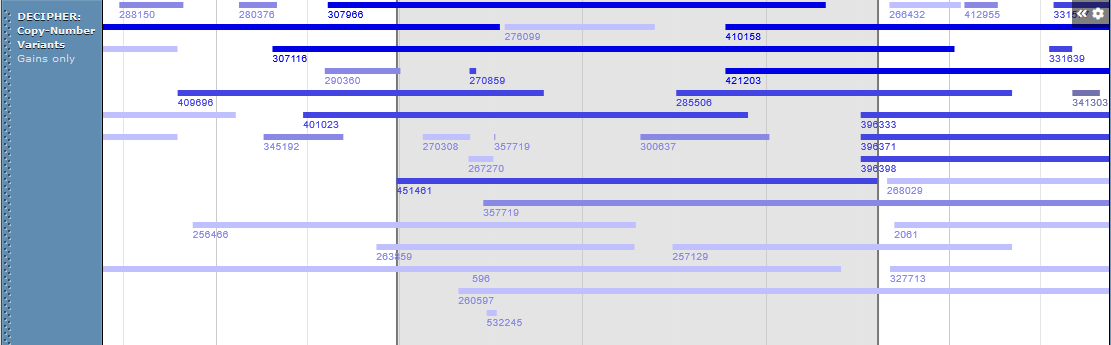


***Figure 4.*** *DECIPHER view Ideogram showing the genes in the 5q14.3q15 region.* *All patients reported in region 5q14.3q15 with copy number gains.*


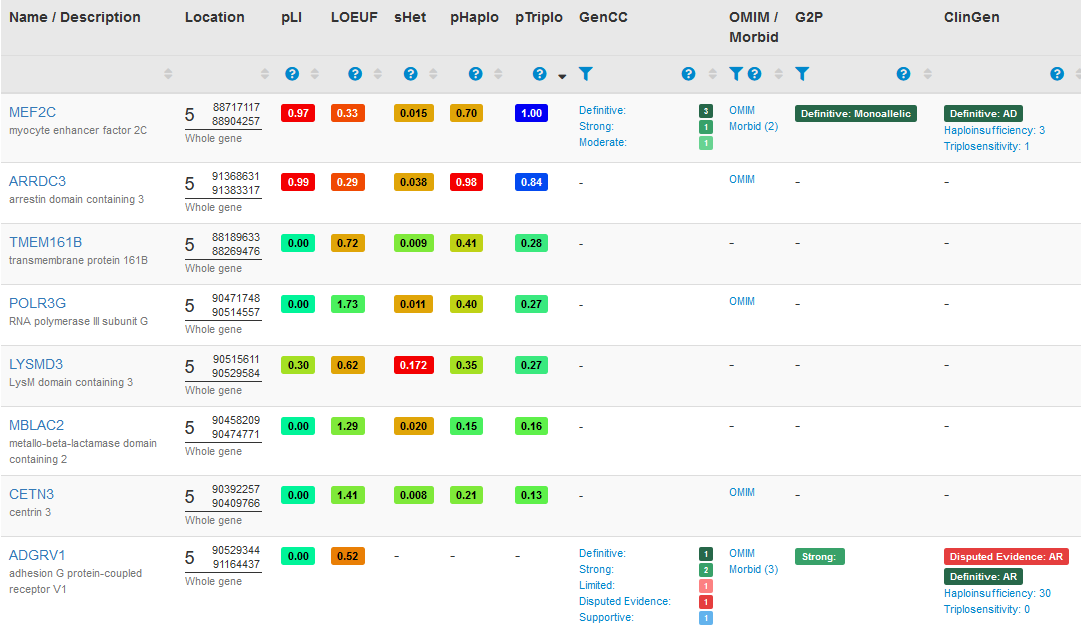


***Figure 5.*** *Dechipher view of region with copy number gain 5q14.3q15 including gene triplosensitivity scores*

Supplementary details regaarding pathogenicity score of the 5q14.3q15 copy number gain was calculated to be 1.2, according to the ClinGen CNV Pathogenicity Calculator (15), which indicates that the detected copy number gain has pathogenic significance in acordance with American College of Medical Genetics and Genomics (ACMG) and the Clinical Genome Resource (ClinGen) 2020 guideline (15).

The score was calculated for the region as follows:

1A. Contains protein-coding or other known functionally important elements **(score 0)**; 2H. Haploinsufficient gene fully contained within observed copy number gain **(score 0);** 3A. Number of protein-coding RefSeq genes wholly or partially included in the copy number gain 0-34 genes **(**30 genes **- score 0)**; 4B. the reported phenotype is consistent with the gene/genomic region, is highly specific, but is not necessarily unique to the gene/genomic region (**score 0.9**, Patient reported by Siddharth Banka et al. (16) Decipher patient ID 596 had anterior segment dysgeensis considered very speicific, while their 5 patients reported in Decipher had global developmental delay, autistic beahviour and,abmormality of growth one also with microcephaly); 5A De novo scoring - confirmed de novo **(Score 0.3).**

The patient's genomic and phenotypic data were reported to Decipher Patient ID 451461 and ClinVar ID 3775074.
